# The role of inflammation and metabolic risk factors in the pathogenesis of calcific aortic valve stenosis

**DOI:** 10.1007/s40520-020-01681-2

**Published:** 2020-09-25

**Authors:** Maddalena Conte, Laura Petraglia, Pasquale Campana, Gerardo Gerundo, Aurelio Caruso, Maria Gabriella Grimaldi, Vincenzo Russo, Emilio Attena, Dario Leosco, Valentina Parisi

**Affiliations:** 1 Department of Translational Medical Sciences, University of Naples Federico II, Via Pansini 5, 8031 Naples, Italy; 2 Casa di Cura San Michele, Maddaloni, Italy; 3 University of Campania Luigi Vanvitelli, Caserta, Italy; 4 San Giuliano Hospital, Naples, Italy

**Keywords:** Epicardial adipose tissue, Aortic stenosis, Inflammation, Aging

## Abstract

Given the epidemiologic increase of aged population in the world, aortic stenosis (AS) represents now the most common valvular heart disease in industrialized countries. It is a very challenging disease, representing an important cause of morbidity, hospitalization and death in the elderly population. It is widely recognized that AS is the result of a very complex active process, driven by inflammation and involving multifactorial pathological mechanisms promoting valvular calcification and valvular bone deposition. Several evidence suggest that epicardial adipose tissue (EAT), the visceral fat depot of the heart, represents a direct source of cytokines and could mediate the deleterious effects of systemic inflammation on the myocardium. Importantly, obesity and metabolic disorders are associated with chronic systemic inflammation leading to a significant increase of EAT amount and to a pro-inflammatory phenotypic shift of this fat depot. It has been hypothesized that the EAT inflammatory state can influence the structure and function of the heart, thus contributing to the pathogenesis of several cardiac diseases, including calcific AS. The current review will discuss the recently discovered mechanisms involved in the pathogenesis of AS, with particular attention to the role of inflammation, metabolic risk factors and pro-fibrotic and pro-osteogenic signal pathways promoting the onset and progression of the disease. Moreover, it will be explored the potential role of EAT in the AS pathophysiology.

## The evolving epidemiology of calcific aortic valve stenosis

Aortic stenosis (AS) is the most common type of valvular heart disease in developed countries and its prevalence draamatically increases with age: in US population, the prevalence of moderate or severe AS ranges from 0.02 to 0.1% in subjects aged 18–44 years and from 2.8 to 4.6% in patients 75 years or older [1]. Similar trends are described in Europe. The EuroHeart Survey evaluated the incidence of valve disease among 92 centres across 25 countries. The mean age of patients with valve disease was 65 ± 14 years and AS was the most common valve disease, occurring in 43.1% of subjects [2]. As life expectancy and the aging population increase, the number of AS patients will dramatically raise. In the European countries, it has been estimated that the number of subjects with symptomatic severe AS will increase from 1.3 million in 2025 to 2.1 million in 2050, due to the demographic shift of industrialized countries. Therefore, AS will have a significant impact on public health and health care resources. In fact, in advanced stages of the disease, AS is associated with functional decline and frequent hospitalizations. Moreover, it is well recognized that untreated, and severe AS is associated with poor prognosis: it has been described an average survival rate of 50% at 2 years after symptoms onset and of 20% at 5 years [3, 4]. Currently, there is no available pharmacological therapy. Indeed, surgical and transcatheter aortic valve replacement (AVR) represent the only therapeutic strategies for AS. Thus, AS early recognition and treatment are imperative. However, the high frequency of comorbidity in the elderly and the increased risk associated with invasive procedures raise important issues on AS management in the elders. To allow the development of new therapeutic strategies for AS, it is very important to understand the complex multifactorial mechanisms involved in the pathogenesis of the disease.

## Active mechanisms involved in pathogenesis of calcific aortic valve stenosis

In the adult population, especially in patients over 70 years, calcific degeneration represents the main mechanism involved in the development of AS.

It is a progressive and potentially fatal disease, that starts with an initial and asymptomatic stage, known as aortic valve sclerosis, characterized by non-uniform leaflet thickening and spotty calcium deposition, with preserved valvular function. Then, valvular degeneration advances to the end-stage of disease, called AS, with severe calcification and obstruction of the left ventricular (LV) outflow. For decades, AV calcification has been thought to be caused by chronic passive and degenerative changes associated with aging. Nowadays, it is widely recognized that AS is a progressive and active process, involving complex and multifactorial pathological mechanisms. AS pathophysiology shares many features with vascular atherosclerosis, thus traditional atherosclerotic risk factors, such as age, smoking, hypertension, hypercholesterolemia, obesity, and diabetes, are also associated with AS development [5]. The calcific degeneration of aortic valve (AV) includes complex mechanisms, such as endothelial dysfunction, oxidative stress, and inflammation.

### Mechanical stress

During the cardiac cycle, AV is subjected to mechanical stresses which play an important role in valve calcification by damaging the structural integrity of the leaflet tissue. This mechanical stress causes damage to valvular endothelial cells and consequent dysfunction of the endothelium which loses the barrier function against metabolic, mechanical, and inflammatory insults. Endothelial injury represents a crucial AS pathophysiological element, allowing lipid and inflammatory cells infiltration into the interstitial valvular tissue.

### Lipid deposition and oxidative stress

Lipid deposition and oxidative stress play a key role in the development of AV degeneration. Similar to the earliest stages of atherosclerotic plaques, the presence of low-density lipoprotein (LDLs) and lipoprotein A has been shown at microscopic analysis in early AV lesions [6]. These molecules undergo oxidative modifications, thus starting the cellular signalling cascade leading to AV calcification [7]. LDLs oxidation is associated with the release of highly cytotoxic free radicals, such as superoxide and oxygen peroxide, and with the secretion of proinflammatory and profibrotic cytokines which promote inflammatory activity and mineralization. Oxidized LDLs also stimulate the valve fibroblasts activity, thus promoting the building of a central core for calcium deposition [6, 7]. The marked increase in oxidative stress shown in stenotic AV, largely due to the impaired function of antioxidant enzymes, triggers the activation of pro-fibrotic and pro-osteogenic signals involved in the pathogenesis and progression of AS [8].

### Inflammation

Monocytes and T lymphocytes are the mainly inflammatory cells involved in the pathogenesis and progression of AS. The increased endothelial expression of adhesion molecules, such as E-selectin, intercellular adhesion molecule-1 (ICAM-1) and vascular cell adhesion molecule-1 (VCAM-1), allows these cells to adhere and infiltrate the valvular sub-endothelium, where they differentiate in macrophages and activated T cells and release growth factors and proinflammatory cytokines such as interleukin-1 (IL-1) and tumor necrosis factor alpha (TNF-α), thus promoting fibrosis and calcification. Local inflammation also stimulates pathological angiogenesis processes contributing to the progression of calcific valve degeneration [9, 10].

### Renin–angiotensin–aldosterone system

Moreover, also the renin–angiotensin–aldosterone system seems to have a role in the pathogenesis of AS, through the increased local expression of the angiotensin II type 1 (AT1) receptor and the increased activity of Angiotensin II, which favours LDL uptake and induces a pro-inflammatory and pro-fibrotic state [11].

### Extracellular matrix remodeling and bone deposition

In the advanced phases of the disease, inflammatory cytokines promote a remodeling of the extracellular matrix by the activation of metalloproteinases and fibroblasts proliferation, inducing fibrosis, thickening and increased valvular stiffness [12]. As the disease progresses, the formation and deposition of bone tissue at the valvular level occur. Typical proteins of bone extracellular matrix, such as osteonectin, osteopontin, and osteocalcin, have also been found in the calcified valves, thus supporting the concept that AV calcification occurs in a similar way to that observed in the bone, starting with an initial deposition of collagen matrix, which provides a basis for progressive calcification [13]. Bone valvular deposition involves the activation of multiple signaling pathways responsible for the differentiation of the valvular interstitial cells, the most abundant cell type in cardiac valves, into myofibroblasts and osteoblast-like cells, which give rise to calcification nodules [14]. Importantly, in stenotic valves, it has been observed an increased expression of osteoblast-specific proteins implicated in skeletal bone formation, such as osteopontin, bone sialoprotein and bone-forming proteins 2 and 4 [15, 16]. In the more advanced stages of the disease, very complex calcified pathways have been implicated in the modulation of osteoblastic differentiation, including the Notch, Wnt/β-catenin, and receptor activator of nuclear factor kappa B/receptor activator of nuclear factor kappa B ligand/osteoprotegerin pathways (RANK/RANKL/OPG) [15, 18, 19]. RANK is a transmembrane protein, whose expression has been demonstrated both in osteoclast precursors and in interstitial valve cells. However, the binding with its ligand, RANKL, a member of the TNF cytokine family, causes opposite effects in bone tissue and in AV. In the bone tissue, RANKL binds to RANK and induces osteoclasts differentiation and maturation, thus promoting bone resorption and demineralization processes. On the other hand, in interstitial valve cells, the same binding of RANKL to RANK strongly promotes osteoblasts differentiation, thus inducing calcification and bone deposition, leading to valve stenosis [20].

## Abdominal visceral adipose tissue and aortic valve calcification

It is known that visceral obesity is associated to insulin resistance and to a cluster of metabolic abnormalities often referred to metabolic syndrome, whose prevalence has significantly increased in the last decades. Interestingly, the increase in visceral fat is associated with a higher incidence of cardiovascular events, including AS [21, 22]. Abdominal visceral fat exerts its deleterious effects through the production and secretion of various proinflammatory cytokines, that promote the development of diabetes mellitus and insulin resistance, which is a fundamental characteristic of metabolic syndrome. The chronic inflammatory state produced by visceral obesity is associated with a pro-atherogenic alteration of the lipid profile, which contributes to increased cardiovascular risk also through an increase in small, low-density lipoproteins [23].

Visceral obesity and metabolic syndrome are strongly associated not only with the development and progression of coronary artery disease, but they also appear to be related to the development of calcific AV degeneration. In this regard, several reports have focused on the role of visceral fat in development of AS. In the Multi-ethnic Study of Atherosclerosis (MESA), metabolic syndrome was found to be associated with a significant increase in incidence of AV calcification, identifying metabolic syndrome as a potential therapeutic target to prevent the disease [24]. More recently, Oikawa et al. explored the relationship between the abdominal visceral adipose tissue and the presence of AV calcification, thus identifying the visceral fat as an independent risk factor for AS [25].

The Aortic Stenosis Progression Observation Measuring Effects of Rosuvastatin (ASTRONOMER) study has reported an association of metabolic syndrome not only with the development of AV calcification, but also with the progression of aortic valve disease [26]. Overall, these data indicate that the visceral adipose tissue could play a role in AS pathogenesis through the inflammatory state and the enhanced oxidative stress, as indicated by the high production of inflammatory cytokines and reactive oxygen species (ROS) in this population. In this regard, both in animal models of obesity and in obese humans, an increased expression of TNF-α, nicotinamide adenine dinucleotide phosphate (NADPH) oxidase and plasminogen activator inhibitor-1 in the adipose tissue has been described [27].

## Epicardial adipose tissue in calcific aortic valve stenosis

### Epicardial adipose tissue (EAT) and inflammation

It is widely recognized that obesity promotes systemic inflammation and exacerbates the inflammatory burden imposed by many chronic extracardiac comorbidities [28]. Importantly, obesity related chronic systemic inflammation is associated to a significant increase in the amount of EAT, the cardiac visceral fat, which is considered a transducer of the adverse effects of systemic inflammation and metabolic disorders on the heart [29]. In physiologic conditions, EAT exerts a protective action for the heart. However, it is known that EAT may also play an unfavorable activity, representing an important source of various pro-inflammatory and pro-atherogenic cytokines, which can negatively affect the myocardium and epicardial coronary arteries through paracrine or vasocrine actions. EAT and the myocardium are tightly connected, without anatomical boundaries, and they share the coronary microcirculation [30]. There is a bidirectional continuum between EAT and systemic inflammation: EAT releases proinflammatory cytokines into the general circulation contributing to the systemic inflammatory state; systemic inflammation, in turn, can induce EAT accumulation and EAT pro-inflammatory phenotype, thus promoting local inflammation and end-organ dysfunction. In this regard, EAT accumulation also results in an increased EAT production and secretion of proinflammatory factors, such as leptin, TNF-α, IL1-β, IL-6, and resistin. In this context, EAT thickness can be considered a specific marker of visceral adiposity [31, 32]. Echocardiographic measurement of EAT thickness well correlates with EAT volume assessed at cardiac magnetic resonance [33].

### EAT and AS

**Fig. 1:**
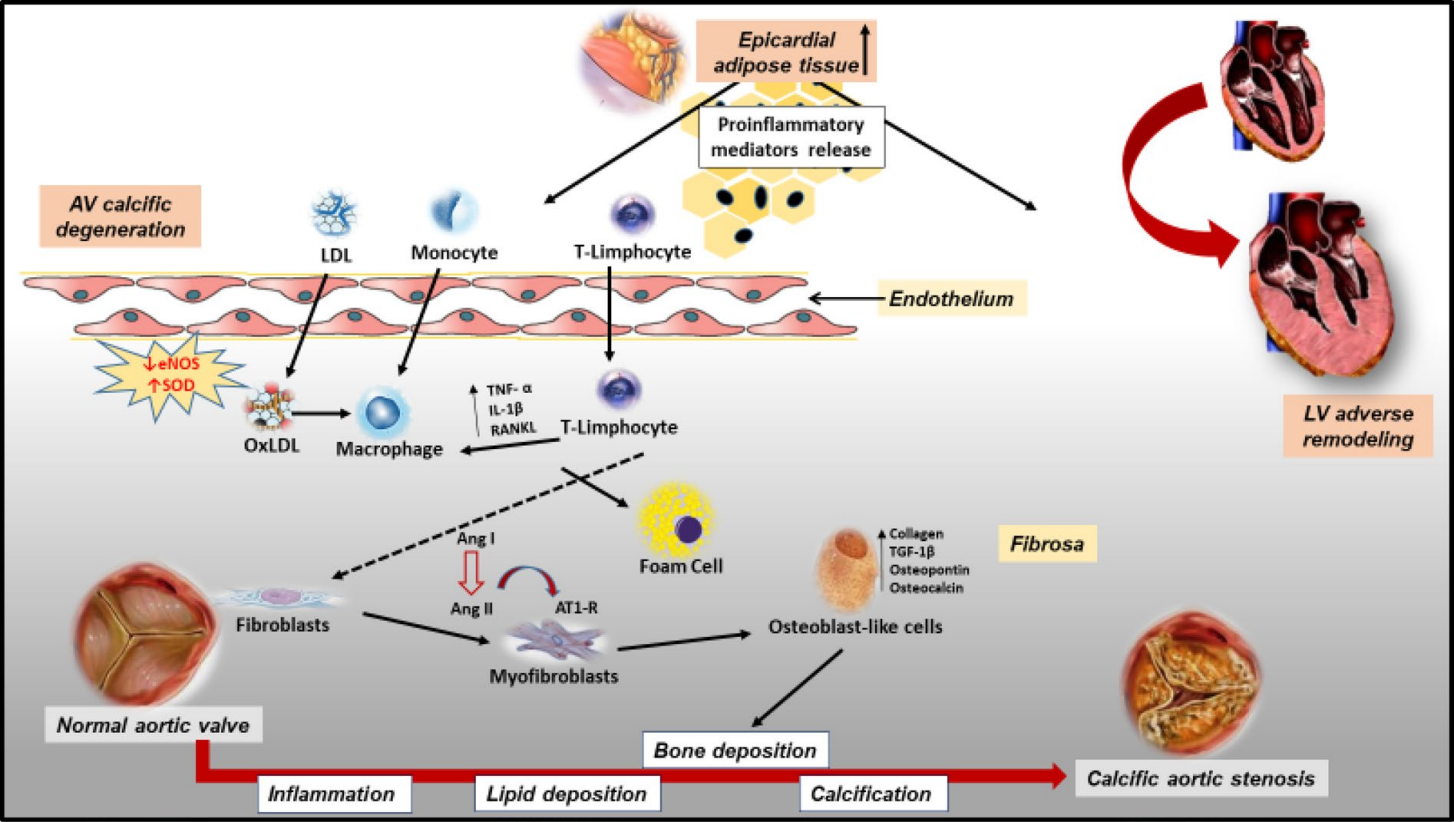
Active mechanisms involved in the pathogenesis of calcific aortic stenosis and the potential involvement of epicardial adipose tissue. Lipid deposition, oxidative stress, and inflammation play a key role in the development and the progression of calcific aortic valve degeneration, allowing circulating macrophages and T lymphocytes to penetrate the endothelium of aortic valve leaftlets. These cells secrete proinflammatory cytokines, such as interleukin 1β (IL-1β), tumor necrosis factor (TNF-a), IL-6, IL-8, insulin-like growth factor 1, transforming growth factor β (TGF-β), thus promoting the differentiation of resident valvular interstitial cells to an osteoblast-like phenotype, leading to bone deposition and calcification. The increase of EAT thickness and its pro-inflammatory status, through the secretion of pro-inflammatory cytokines may promote these inflammatory and atherogenic phenomena occurring in the aortic valve; moreover, EAT pro-inflammatory activation may drive LV hypertrophy and fibrosis promoting adverse remodeling

Several studies explored whether EAT could contribute to the inflammatory burden of AS. To this aim, Parisi et al. reported an EAT increased echocardiographic thickness in patients with AS. Of interest, EAT thickness significantly correlated with the levels of different EAT-derived pro-inflammatory and pro-atherogenic cytokines, such as IL-6, TNF-α, MCP-1, and IL-1β. These results support the hypothesis of a potent pro-inflammatory activation of EAT in patients with AS and suggest the involvement of cardiac visceral fat in inflammatory and atherogenic phenomena occurring in the AV and promoting its degeneration and calcification [34] (Fig. 1).

A retrospective study conducted by Mahabadi et al., included a cohort of 200 consecutive patients with severe AS and 200 matched patients without AS. EAT thickness, assessed by transthoracic echocardiography, was significantly higher in AS patients and the association remained stable after adjustment for age, gender, and traditional cardiovascular risk factors. These data confirm the strong association between EAT thickness and severe AS, independently of traditional risk factors [35]. A more recent prospective study evaluated the impact of EAT (assessed by a multi-detector computed tomography) on outcome, in 503 consecutive AS patients undergoing transcatheter aortic valve replacement (TAVR). EAT volume was independently associated with all-cause mortality at 1, 2, and 3 years after TAVR, thus identifying EAT as an important prognostic factor in this population [36]. Similarly, EAT assessed at cardiac magnetic resonance can predict prognosis and symptoms onset in AS patients [37]. The association between EAT, symptoms, and prognosis probably lie in the EAT-related left ventricular (LV) unfavorable remodeling. At this regard, it is known that pressure overload determined by AV narrowing induces an LV remodeling up to concentric hypertrophy, in attempt to offset the raised intracavitary pressure associated with AS and to allow the heart to maintain adequate cardiac output. However, as the disease progresses, this response becomes a maladaptive phenomenon and evolves towards diastolic dysfunction, finally leading to heart failure [38]. A close correlation between an increase in EAT and increase in LV mass has been described in literature and supports the role of EAT in promoting myocardial hypertrophy [39]. The specific association between EAT echocardiographic thickness and LV remodeling has been recently explored by a prospective study including 202 consecutive patients with severe AS and preserved LV ejection fraction, referred to surgical AVR. In this population, increased EAT was associated with LV hypertrophy and adverse remodeling. Therefore, increased EAT thickness could result in enhanced hypertrophic stimuli induced by chronic pressure overload and contribute to negative cardiac remodeling [40].

Noteworthy, all the studies cited above suffer the limitation of reporting only the association between EAT and AS. Further studies, also conducted in preclinical AS models, are needed to establish a causative mechanism between structural and functional modifications of EAT and the development of aortic valve degeneration and its progression.

## Conclusion

Calcific AS represents the most common valvular heart disease and one of the major cause of morbidity and mortality in the elderly. Its pathogenesis involves a very complex process that advances in several stages characterized by lipid infiltration, neoangiogenesis, endothelial dysfunction and bone deposition. Beyond traditional risk factors, inflammation and metabolic disorders may actively contribute to the development and progression of the disease. Obesity and metabolic disorders are associated to a chronic systemic inflammation and to a significant increase of the EAT amount, the visceral fat surrounding the heart, that is accompanied by a pro-inflammatory status of this fat depot. Several evidence suggest that EAT, through the production and release of proinflammatory cytokines, can influence the structure and function of the heart, thus contributing to the pathogenesis of several cardiac diseases, including calcific AS.

A better understanding of the biological active mechanisms promoting calcification and bone deposition at the aortic valve level, related to inflammation and metabolic risk factors, will help to define tailored therapeutic strategies for the treatment and prevention of the disease in this setting of high-risk patients.
